# Polymorphisms of vascular endothelial growth factor on prognosis in osteosarcoma patients

**DOI:** 10.12669/pjms.305.5170

**Published:** 2014

**Authors:** Zhao Dong-ju, Xiao Ai-ju, Tian Yun-jiao, Zhang Ming-qiu

**Affiliations:** 1 Zhao Dong-ju, Department of Pediatrics, The First Affiliated Hospital of Xinxiang Medical University, Weihui 453100, China.; 2 Xiao Ai-ju, Department of Pediatrics, The First Affiliated Hospital of Xinxiang Medical University, Weihui 453100, China.; 3 Tian Yun-jiao, Department of Pediatrics, The First Affiliated Hospital of Xinxiang Medical University, Weihui 453100, China.; 4 Zhang Ming-qiu, Department of Invasive Technology, The First Affiliated Hospital of Xinxiang Medical University, Weihui 453100, China.

**Keywords:** Vascular endothelial growth factors; osteosarcoma; chemotherapy; clinical outcome

## Abstract

***Objective: ***We conducted a cohort study to investigate the association of three common SNPs of vascular endothelial growth factors (VEGF) gene (+1612G/A, -634C/G and +936G/C) with clinical outcome of osteosarcoma in a Chinese population.

***Methods:*** A prospective study was conducted. Genotyping analyses of VEGF -2578C/A, +1612G/A, -634C/G and +936G/C were conducted using polymerase chain reaction-restriction fragment length of polymorphism. Multivariate Cox proportional hazards models were used to calculate hazard ratio (HR) and 95% CI of effect of each genotype of VEGF+1612G/A, -634C/G and +936G/C on PFS and osteosarcoma of osteosarcoma.

***Results: ***The good response rate was 52.29%, and 116 (68.7%) died during the follow-up period. Patients carrying the +936 CC genotype and C allele showed a significantly more response to chemotherapy than those carrying the wild-type genotype. In the Cox proportional hazards model, patients carrying the VEGF -634 T allele was associated with a significantly decreased risk of PFS and Osteosarcoma (OS). Patients carrying the +936 CC genotype and C allele were associated with a significantly decreased risk of presenting progressive disease or death from osteosarcoma when compared with those carrying the wild-type genotype. However, we observed no significant association between the VEGF -2578C/A and +1612A/G polymorphisms and PFS and Osteosarcoma (OS) in gastric cancer patients.

***Conclusions:*** We found that VEGF -634G/C and +936T/C polymorphisms may affect the prognosis of osteosarcoma patients. These finding may be useful for predicting the clinical outcome of patients with Osteosarcoma (OS). Further studies are greatly needed to confirm the clinical significance of these results.

## INTRODUCTION

Osteosarcoma(OS) is the most frequent primary malignant bone tumor in children and adolescents, which accounts for 0.2% of all cancer and 20% of all primary sarcomas in bone.^1^^-^^3^ Despite recent advances in the therapies, the overall survival for patients with osteosarcoma is still low and unsatisfactory.^4^ Osteosarcoma patients with metastatic disease usually showed a shorter survival time.^5^ It is reported that 40% of these patients present a poor response to chemotherapy, and the five-year survival rate of localized osteosarcoma is usually about 60-80%, while metastatic disease had a poorer prognosis. ^6^^,^^7^ Osteosarcoma patients with similar tumor stages presented different overall survival time and response to chemotherapy. Therefore, cumulative evidences indicated that genetic factors may influence chemotherapy toxicity and clinical outcome of osteosarcoma.^8^^-^^10^

The vascular endothelial growth factors (VEGF) gene is one of the most potent endothelial cell mitogenes, and this gene consists of 8 exons to form a family of proteins.^11^ It is reported that 30 kinds of single nucleotide polymorphisms (SNPs) existed in VEGF gene.^12^ Three common SNPs, VEGF +1612G/A, -634C/G and +936G/C, are widely investigated, and are significantly associated with VEGF protein production.^13^

Several previous studies have indicated that over expression of VEGF is associated with biomarkers of prognosis of patients with osteosarcoma.^14^^-^^16^ However, only one study reported the association between VEGF polymorphisms and prognosis of osteosarcoma in Chinese population.^17^ Therefore, we conducted a cohort study to investigate the association of three common SNPs of VEGF gene (+1612G/A, -634C/G and +936G/C) with clinical outcome of osteosarcoma in a Chinese population.

## METHODS

The study population comprised of 262 diagnosed osteosarcoma patients between September 2007 and September 2009 at the First Affiliated Hospital of Xinxiang Medical University. All cases were newly diagnosed and histologically confirmed with primary osteosarcoma. The written informed consent was obtained from all osteosarcoma patients before participating into study. The protocol of our study was approved by the ethics committee of the First Affiliated Hospital of Xinxiang Medical University.

Osteosarcoma patients received preoperatively with intravenous 25-30 mg/m^2 ^doxorubicin for three days, 14 mg/m^2 ^methotrexate for one day and 35 mg/m^2 ^cisplatin for three days. After receiving surgery, osteosarcoma patients received adjuvant chemotherapy and the regimen included10 mg/m^2^ methotrexate for one day, 0.45 mg/m^2^ cisplatin and 1.5 mg/m^2^ vincristine for one day, and 500mg/m^2^ cyclophosfamide for three days. The treatment was repeated every three weeks for a maximum of six cycles. The toxicity assessment was conducted before each cycle. The treatment would not be continued when patient presented with progressive disease or experienced unacceptable toxicity. If osteosarcoma patients showed three or four grades of hematology toxicity, the chemotherapy dosage was reduced by 25% in the next cycle.

The response to chemotherapy was classified by the response evaluation criteria from European Organization for Research and Treatment of Cancer. The response to chemotherapy was assessed after receiving treatment, and divided into good responders and poor responders. Progression-free survival (PFS) was calculated from the date of enrolling in this study to the date of progressive disease or death. Overall survival (OS) was calculated from the date of enrolling in this study to the date of death or last clinical follow-up.

All the patients were followed up until 30^th^ September 2012, with a median follow-up time of 36.3 months (ranged: 2 to 60 months). All patients were followed up by telephone every four weeks until death or the end of study.

***DNA extraction and PCR amplication*** *: *All study subjects were asked to provide 5 ml peripheral venous blood. According to the manufacturer’s instructions, genomic DNA was extracted from peripheral venous blood samples using the TIANamp blood DNA kit (Tiangen Biotech, Beijing, China). Genotyping analyses of VEGF +1612G/A, -634C/G and +936G/C were detected by polymerase chain reaction-restriction fragment length of polymorphism (PCR-RFLP). The primers used for VEGF -2578C/A, -1154G/A, -634C/G and +936G/C were designed using Sequenom Assay Design 3.1 software (Sequenom®) according to the manufacturer instructions. The cycling programme involved preliminary denaturation at 95 °C for 5 min, followed by 35 step cycles of denaturation at 95 °C for 30s, annealing at 62 °C for 30 s, 72°C for 30s, and a final extension at 72 °C for 10  min. For quality control, 5% of subjects were randomly selected, and the results of repeated samples showed 100% concordance.

***Statistical analysis*** *: *Continuous variables are shown by mean ± standard deviation (SD), while categorical variables are expressed as frequencies and percentages (%). The odds ratios (OR) and corresponding 95% confidence intervals (CIs) were calculated by unconditional logistic regression analysis and utilized to assess the potential association between genotypes frequencies and response to chemotherapy of OS patients. Homozygotes of the most frequent genotype were regarded as reference group. Hazard ratio (HR) and 95% CI were calculated by multivariate The Cox proportional hazards models and used to evaluate the effect of VEGF +1612G/A, -634C/G and +936G/C polymorphisms on PFS and OS of OS. All *P* values were two-tailed, and differences were considered statistically significant when *P*<0.05. SPSS® statistical package, version 11.0 (SPSS Inc., Chicago, IL, USA) for Windows® was used for statistical analyses.

## RESULTS

The distributions of selected general characteristics of study subjects are shown in Table-I. The mean age of the osteosarcoma subjects were 24.6±8.2 years old (ranging 10 to 35 years old). Of 262 OS patients, 163 (62.1%) were males, and 153 (58.4%) presented tumor location at lower limb, 98 (37.5%) showed metastasis at diagnosis, and 152 (57.9%) received Limb salvage. At the end of follow-up, 116 (68.7%) died.

Total 137 patients showed good response to chemotherapy, with a response rate of 52.29% (Table-I). Patients were classified into good and poor responders, and a significantly different genetic distribution of VEGF +936T/C was observed between these groups. Patients carrying the +936 CC genotype and C allele showed a significant more good responder than those carrying the wild-type genotype, with ORs (95% CI) of 7.23(2.28-29.96) and 2.17(1.44-3.30), respectively. 

However, we observed no significant between-group differences in the frequencies of VEGF +1612A/G and -634G/C.

In the Cox proportional hazards model, after adjusting for potential confounding factors, the HRs (95%CI) for PFS and OS in patients carrying the VEGF -634 T allele respectively were 0.48(0.31-0.72) and 0.66(0.45-0.97) using the G allele as the reference variable (Table-III). 

Patients carrying the VEGF +936 CC genotype and C allele were associated with a significantly decreased risk of presenting progressive disease, and the HRs (95%CI) for PFS were 0.32(0.13-0.81) and 0.36(0.12-0.96), respectively. Moreover, VEGF +936 CC genotype and C allele carriers were related to a significantly decreased risk of death from OS, and the HRs (95%CI) for OS were 0.56(0.37-0.85) and 0.63(0.42-0.95), respectively. By Kaplan-Meier method, we found that those carrying VEGF +936 CC genotype and C allele carriers had significantly shorter PFS and OS time when compared with TT genotype (Fig. 1 and 2).

However, we observed no significant association between the VEGF -2578C/A and +1612A/G polymorphisms and PFS and OS in gastric cancer patients.

## DISCUSSION

VEGF, a growth factor that regulates angiogenesis, is regarded to be the most potent stimulatory cytokine regulating tumor angiogenesis and an important factor in metastasis, survival, and tumor spread.^18^ In this study, we observed that the VEGF-634CC and +936CC genotype were correlated with a better response to chemotherapy and a longer survival time in OS patients.

**Table-I:** General characteristics of osteosarcoma subjects

**Age at diagnosis, y **	**Patients, N**	**%**
Median (range)	24.6±8.2	
≤20	140	53.44
＞20	122	46.56
Sex		
Male	163	62.21
Female	99	37.79
*Tumor location*		
Upper limb	109	41.60
Lower limb	153	58.40
*Metastasis*		
Yes	98	37.40
No	164	62.60
*Therapy*		
Amputation	110	41.98
Limb salvage	152	58.02
*Death*		
No	146	55.73
Yes	116	44.27
*Chemotherapy*		
Good	137	52.29
Poor	125	47.71

**Table-II:** VEGF polymorphisms association with tumor response to chemotherapy

***Genotype***		***Patients N=262***	***%***	***Tumor response***				***OR(95%CI)*** ^1^	***P value***
				***Good***	***%***	***Poor***	***%***		
+1612A/G	GG	93	35.50	47	34.31	46	36.80	1.0(Ref.)	-
	GA	119	45.42	64	46.72	55	44.00	1.14(0.64-2.03)	0.64
	AA	50	19.08	26	18.98	24	19.20	1.06(0.50-2.24)	0.86
	A allele	305	58.21	158	115.33	147	117.60	1.0(Ref.)	-
	G allele	219	41.79	116	84.67	103	82.40	1.05(0.73-1.51)	0.79
-634G/C	CC	96	36.64	46	33.58	50	40.00	1.0(Ref.)	-
	CT	137	52.29	73	53.28	64	51.20	1.24(0.71-2.16)	0.42
	TT	29	11.07	18	13.14	11	8.80	1.78(0.70-4.62)	0.18
	C allele	288	54.96	165	120.44	164	131.20	1.0(Ref.)	-
	T allele	195	37.21	109	79.56	86	68.80	1.26(0.87-1.83)	0.2
+936T/C	TT	140	53.44	62	45.26	78	62.40	1.0(Ref.)	-
	TC	95	36.26	52	37.96	43	34.40	1.52(0.87-2.66)	0.12
	CC	27	10.31	23	16.79	4	3.20	7.23(2.28-29.96)	<0.001
	T allele	375	71.56	176	128.47	199	159.20	1.0(Ref.)	-
	C allele	149	28.44	98	71.53	51	40.80	2.17(1.44-3.30)	<0.001

1. Adjusted for sex, age, tumor location, metastasis and therapy.

**Table-III:** Cox regression analysis of VEGF polymorphisms with the PFS and OS of osteosarcoma patients

**Genotype**		**PFS**		**HR (95%CI)** ^1^	**P value**	**OS**		**HR (95%CI)** ^1^	**P value**
		**Event**	**%**			**Event**	**%**		
+1612A/G	GG	66	38.60	1.0(Ref.)	-	45	38.79	1.0(Ref.)	-
	GA	76	44.44	0.72(0.39-1.35)	0.28	51	43.97	0.8(0.45-1.43)	0.42
	AA	29	16.96	0.56(0.25-1.24)	0.12	20	17.24	0.71(0.33-1.51)	0.34
	A allele	208	60.82	1.0(Ref.)	-	141	60.78	1.0(Ref.)	-
	G allele	134	39.18	0.74(0.50-1.08)	0.1	91	39.22	0.83(0.57-1.19)	0.29
-634G/C	CC	68	39.77	1.0(Ref.)	-	45	38.79	1.0(Ref.)	-
	CT	86	50.29	0.69(0.38-1.26)	0.2	59	50.86	0.86(0.49-1.50)	0.56
	TT	17	9.94	0.58(0.23-1.53)	0.22	11	9.48	0.69(0.27-1.75)	0.4
	C allele	222	64.91	1.0(Ref.)	-	149	64.22	1.0(Ref.)	-
	T allele	120	35.09	0.48(0.31-0.72)	<0.001	81	34.91	0.66(0.45-0.97)	0.03
+936T/C	TT	100	58.48	1.0(Ref.)	-	69	59.48	1.0(Ref.)	-
	TC	59	34.50	0.66(0.36-1.19)	0.13	40	34.48	0.75(0.43-1.31)	0.28
	CC	12	7.02	0.32(0.13-0.81)	0.006	7	6.03	0.36(0.12-0.96)	0.03
	T allele	259	75.73	1.0(Ref.)	-	178	76.72	1.0(Ref.)	-
	C allele	83	24.27	0.56(0.37-0.85)	0.004	54	23.28	0.63(0.42-0.95)	0.02

1. Adjusted for sex, age, tumor location, metastasis and therapy.

The VEGF gene is located at chromosome 6p21.3 and consists of 8 exons. It is estimated that there are more than 30 single-nucleotide polymorphisms (SNPs) in VEGF. DNA sequence variations in the VEGF gene may alter VEGF production and/or activity, thereby causing interindividual differences in susceptibility to cancer because of their effects on tumor angiogenesis pathways.

For OS, only one previous study has reported that +936C>T has been reported to be significantly associated with VEGF protein production.^18^ Our study reported a similar result that -634G/C and +936C>T polymorphisms play a major role in the clinical outcome of OS. It is reported that potential functional SNPs of VEGF (-634G/C and +936C>T) could affect the protein translation efficiency, circulating plasma concentrations and tumor tissue expression of VEGF.^19^^-^^21^ Previous studies have showed that -634G/C and +936C>T polymorphisms are associated with clinical outcome of several solid tumors, such as prostate, breast, gastric cancer.^22^ One study conducted in Italy has shown that -634CC genotype was associated with a shorter PFS in prostate cancer patients, but no association between +936C>T polymorphism and PFS of patients.^23^ Another study reported no association between -634G/C polymorphism and survival of gastric cancer.^24^ The inconsistent role of these results may be induced by its association with other functional SNPs in the VEGF gene or other SNPs of the angiogenesis pathway. Moreover, discrepancies in population, case selection, sample size may also cause the difference between results of studies. Further studies are needed to elucidate the potential role of VEGF polymorphisms tumor biology.

There were several limitations in our study. First, cases were selected from one hospital, which may not be representative of the general population. Second, other genetic polymorphisms of the angiogenesis pathway may influence the prognosis of OS besides the *VEGF* gene. Therefore, further large sample, multicenter studies including different ethnicities are warranted to investigate the role of *VEGF* gene polymorphisms on the prognosis of OS.

In conclusion, we found that VEGF -634G/C and +936T/C polymorphisms may affect the prognosis of OS patients. These finding may be useful for predicting the clinical outcome of patients with OS. Further studies are needed to confirm the clinical significance of these results.

## Authors Contributions:

***ZDJ & XAJ: ***Designed and performed the study, and did statistical analysis & editing of manuscript.

***ZDJ, XAJ, TYJ & ZMQ: ***Did data collection and manuscript writing.
